# Atlas of phytoplankton phenology indices in selected Eastern Mediterranean marine ecosystems

**DOI:** 10.1038/s41598-024-60792-2

**Published:** 2024-04-30

**Authors:** Antonia Kournopoulou, Katerina Kikaki, Ioanna Varkitzi, Stella Psarra, Georgia Assimakopoulou, Konstantinos Karantzalos, Dionysios E. Raitsos

**Affiliations:** 1 Department of Biology, National and Kapodistrian University of Athens, 157 72 Athens, Greece; 2 Remote Sensing Laboratory, National Technical University of Athens, 15780 Zographou, Greece; 3 Hellenic Centre for Marine Research (HCMR), Institute of Oceanography, 19013 Anavyssos, Greece; 4 Hellenic Centre for Marine Research (HCMR), Institute of Oceanography, 71003 Crete, Greece

**Keywords:** Ocean sciences, Ecology

## Abstract

Phytoplankton is a fundamental component of marine food webs and play a crucial role in marine ecosystem functioning. The phenology (timing of growth) of these microscopic algae is an important ecological indicator that can be utilized to observe its seasonal dynamics, and assess its response to environmental perturbations. Ocean colour remote sensing is currently the only means of obtaining synoptic estimates of chlorophyll-a (a proxy of phytoplankton biomass) at high temporal and spatial resolution, enabling the calculation of phenology metrics. However, ocean colour observations have acknowledged weaknesses compromising its reliability, while the scarcity of long-term in situ data has impeded the validation of satellite-derived phenology estimates. To address this issue, we compared one of the longest available in situ time series (20 years) of chlorophyll-a concentrations in the Eastern Mediterranean Sea (EMS), along with concurrent remotely-sensed observations. The comparison revealed a marked coherence between the two datasets, indicating the capability of satellite-based measurements in accurately capturing the phytoplankton seasonality and phenology metrics (i.e., timing of initiation, duration, peak and termination) in the studied area. Furthermore, by studying and validating these metrics we constructed a satellite-derived phytoplankton phenology atlas, reporting in detail the seasonal patterns in several sub-regions in coastal and open seas over the EMS. The open waters host higher concentrations from late October to April, with maximum levels recorded during February and lowest during the summer period. The phytoplankton growth over the Northern Aegean Sea appeared to initiate at least a month later than the rest of the EMS (initiating in late November and terminating in late May). The coastal waters and enclosed gulfs (such as Amvrakikos and Maliakos), exhibit a distinct seasonal pattern with consistently higher levels of chlorophyll-a and prolonged growth period compared to the open seas. The proposed phenology atlas represents a useful resource for monitoring phytoplankton growth periods in the EMS, supporting water quality management practices, while enhancing our current comprehension on the relationships between phytoplankton biomass and higher trophic levels (as a food source).

## Introduction

The microscopic algae, phytoplankton, found in nearly every aquatic environment, are vital for the functioning of marine ecosystems. These microorganisms are responsible for $\sim$50% of Earth’s oxygen, serve as the underlying energy source of aquatic ecosystems^1^, and constitute the base of marine food webs, providing an essential food source for other organisms^2^. Phytoplankton also support fisheries resources, providing food security and economic support for maritime nations bordering the Mediterranean^3,4^. Spatiotemporal variations in phytoplankton abundance and timing of growth (phenology) can have a significant impact on marine ecosystems, as the survival and fitness of other organisms in the food web depend on food availability^5–7^. Therefore, monitoring the trends and variability of these ecological indicators can provide important knowledge of ecosystem status.

Ecological indicators, such as phytoplankton abundance and phenology (timing of periodic growth events), can be used to monitor seasonal phytoplankton dynamics, describe their response to environmental/climate change, as well as the transfer of organic carbon to higher trophic levels^5–9^. Phenology metrics include: the timing of the initiation, peak, termination and duration of phytoplankton blooms. The knowledge gained from these indices is highly important for assessing the linkages between phytoplankton and fisheries stocks^5,10^, thus supporting and improving the marine management strategies^11^.

Ocean colour satellite sensors have been widely used for the effective monitoring of phytoplankton ecological indicators; an asset for detecting marine ecosystem changes^12^. Several studies have investigated the interannual variability and trends of phytoplankton at global or basin-wide scales utilizing satellite ocean colour datasets^13–18^. For instance, using satellite-derived observations of ocean colour, evidence has shown that phytoplankton biomass (as indexed by Chlorophyll-a [Chl-a])^19^, influences the survival/hatch rates of several marine organisms (including fish and shrimps)^5,20,21^. To describe the phenology of phytoplankton growth in the global oceans, Racault et al.^22^ analyzed its variability between 1998 to 2007, highlighting the importance of phenology as an indicator for marine resources management. In the Eastern Mediterranean Sea (EMS), a prominent biodiversity hotspot^23,24^—the average duration of phytoplankton growth periods has exhibited a decreasing trend from 1998 to 2014^18^. Such large-scale studies explore overall trends in phytoplankton phenology over broad areas, however, detailed information at regional scales is usually limited.

To successfully retrieve phytoplankton phenology metrics there is a need for continuous long-term, gap-free, datasets. In situ measurements are usually sparse in space and time, making it difficult to apply phenology algorithms and calculate seasonal phytoplankton growth metrics^10^. As an alternative, satellite-derived ocean colour observations are freely available and provide synoptic observations of Chl-a concentrations at high spatial (1 km) and temporal (daily) resolution, which could ultimately support such an approach. However, one of the issues derived, is the actual validity of such metrics based on satellite-derived observations^22^. Validating the ocean colour phenology trends with in situ datasets is particularly challenging, mainly due to the lack of continuous in-water observations in space and time^7^. Regardless of the importance of phytoplankton phenology and the availability of satellite-derived information, there is no detailed information on the seasonal cycles of phytoplankton in the EMS, including open and coastal waters and the semi-enclosed Gulfs of the Ionian and Aegean Seas.

In this study, multi-temporal satellite data and phenology monitoring techniques were utilized to explore the phytoplankton growth timing over the EMS. First, we applied a phenology algorithm, based on the threshold criterion approach^22,25^, on both satellite-derived ocean colour observations and a long-term in situ Chl-a time series acquired from the Saronikos Gulf (> 30 years available), which is well-distributed in time (i.e., relatively gap-free) and the longest time series of Chl-a in the EMS. A detailed atlas of phytoplankton phenology in the EMS is provided, based on long-term ($\sim$23 years), high resolution (1 km) satellite-derived ocean colour datasets. In addition, spatial phenological analyzes were conducted and the seasonal cycles of phytoplankton biomass in several regions within the Ionian, Aegean and Levantine Seas are discussed. Finally, we discuss the seasonal succession of phytoplankton biomass in relation to the regional environment and physical forcing.

## Results and discussion

This part is organized into several subsections including the comparison of satellite-derived and in situ phenology metrics, an Atlas of phenology indices over the EMS, and the analysis of seasonal patterns in several coastal and open regions.

### Seasonal cycles and comparison between satellite-derived and in situ phenology

**Figure 1:**
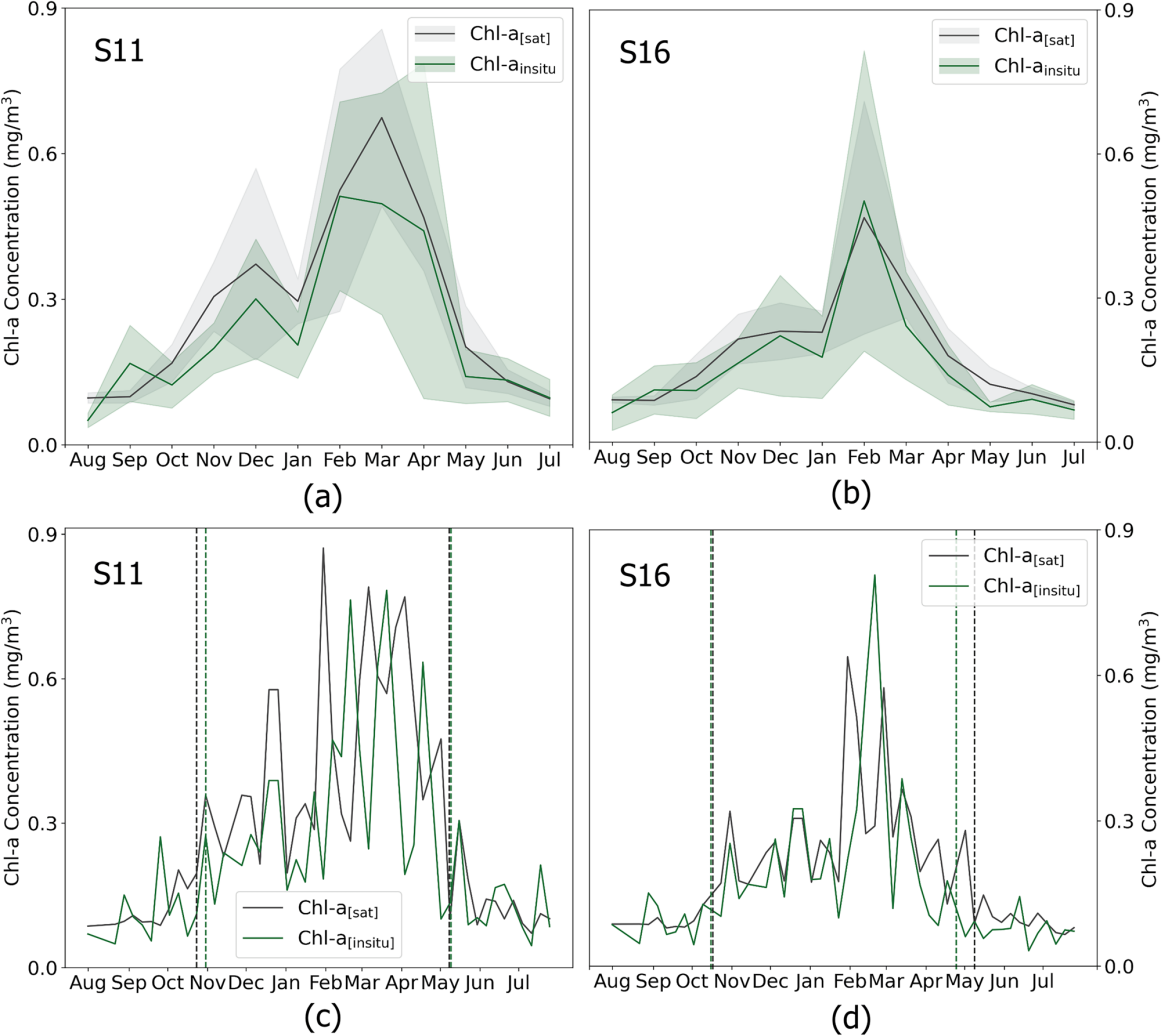
Seasonal Analysis of Chl-a Concentrations in Saronikos Gulf (1997–2017): Seasonal Variations and Phenology. (**a**) Monthly Seasonal Cycle at Station S11, (**b**) Monthly Seasonal Cycle at Station S16, (**c**) Weekly Time Series and Phenology at Station S11, (**d**) Weekly Time Series and Phenology at Station S16. Shaded areas in panels (**a**) and (**b**) represent the 95% confidence interval. Vertical dashed lines in panels (**c**) and (**d**) indicate the initiation and termination of growth periods at both stations.

Monthly seasonal cycles were generated (1a and b), aligning both in situ (Chl-a_[Insitu]_) and satellite (Chl-a_[Sat]_) datasets in terms of spatial and temporal dimensions (as described in the Methods section). In the coastal region of Saronikos Gulf, an increase in light and temperature, coupled with the transfer of nutrients from deeper layers to the illuminated surface zone, leads to typical phytoplankton growth during late winter and spring^26,27^. Both in situ and satellite datasets captured a less intense peak in Chl-a concentration during December and a slight decline in January at the two stations (Fig. 1a and b). This secondary Chl-a peak observed at both stations corroborates findings from previous studies^26,28^. Although both Station S11 and Station S16 exhibit similar Chl-a levels during the blooming period, the latter is generally characterized as more oligotrophic than the former, as evidenced by satellite and in situ observations. Specifically, the *t*ime series data indicate an average Chl-a concentration of 0.24 mg/m^3^ at Station S11 and 0.17 mg/m^3^ at Station S16. This difference is still evident to the present day, with coastal Station S11 transitioning from mesotrophic to oligotrophic conditions since 2005. This shift signifies an improved ecological status, likely due to the implementation of secondary sewage treatment on Psittalia Island^27^.

In order to evaluate the efficiency of satellite-derived phytoplankton phenology, we conducted a direct comparison between phenology metrics calculated using satellite-derived data and in situ datasets in the Saronikos Gulf, over the period 1997–2017 (Fig. 1c and d). Daily matchups were resampled into 7-day bins, and phenology metrics were estimated for both the in situ and satellite datasets following the methods outlined in our study. Phenology indices derived from the two datasets match remarkably well in both stations. According to the (Chl-a_[Insitu]_) time series in S11, the blooming period initiates in late October and terminates in the beginning of May. Satellite-derived phenology in S11 is as the initiation is estimated to occur a week earlier and the termination on the same week (Fig. 1c). Similar results are reported when comparing the phenology metrics at the offshore station S16, where the initiation is identical (i.e. mid-October) and the termination occurs late April and early May, according to (Chl-a_[Insitu]_) and (Chl-a_[Sat]_) time series (Fig. 1d).

### Atlas of phenology metrics

**Figure 2:**
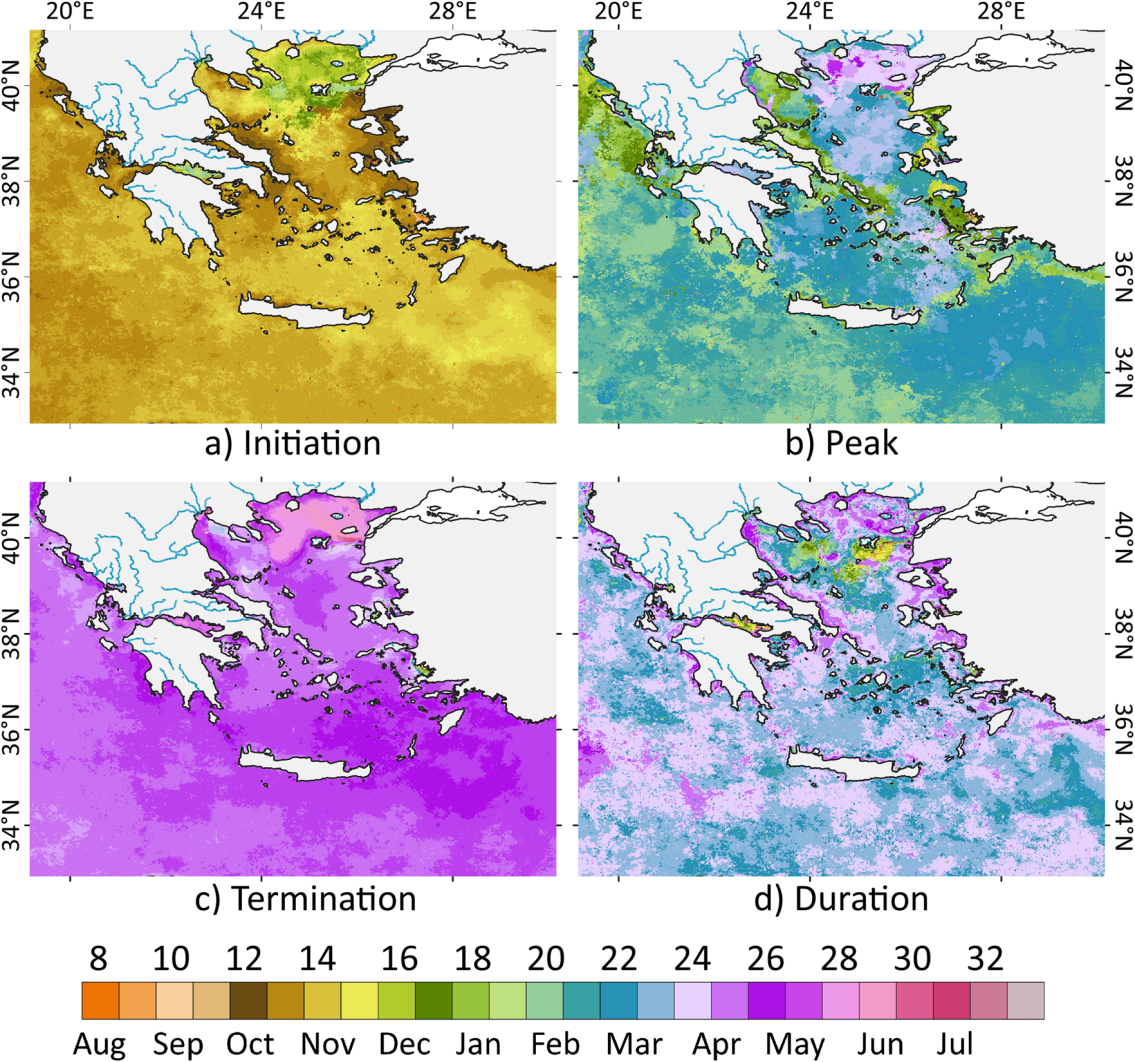
Phenological stages of the main phytoplankton growth in the EMS (based on 23-year climatology of daily composites); (**a**) timing of initiation, (**b**) timing of peak, (**c**) timing of termination, (**d**) duration of the growth period. For panels (**a**), (**b**) and (**c**), the colour scale illustrates the time (as shown below the colour scale) For panel (**d**), the colour scale illustrates the number of 7-day periods (as shown above the colour scale) between the initiation and termination.(Plots created using QGIS 3.16 software, https://www.qgis.org/en/site/index.html).

The EMS exhibited a distinct seasonal pattern with the higher production phase occurring from November to April, characterized by elevated concentrations of Chl-a, with maximum levels observed in February in most open ocean areas. Conversely, the period from May to October exhibits a low production phase^29^. The N. Aegean Sea displays a delayed blooming period compared to the rest of the study area, initiating in December and ending between late May and early June (Fig. 2a and c, respectively). In most coastal regions of EMS, the growth period of phytoplankton begins in mid-October, ending around November (Fig. 2a), slightly earlier compared to the open sea. The peak of the blooming period typically occurs in the middle of winter in most coastal regions, with the highest signal observed from January–March, while in the open sea, Chl-a reaches maximum values during late winter to early spring^30,31^. This gradient of blooming periods creates sub-regions with specific characteristics, which separates the Aegean Sea from north to south. In the Ionian Sea, this distinction is recognized between the coastal zone and the open sea (Fig. 2b). The growth period in the open sea ranges from 23 to 27 weeks (Fig. 2d) and is relatively stable. The subsequent sections will present further analysis of the seasonal cycles and cross-validation for various regions of the EMS.

**Figure 3:**
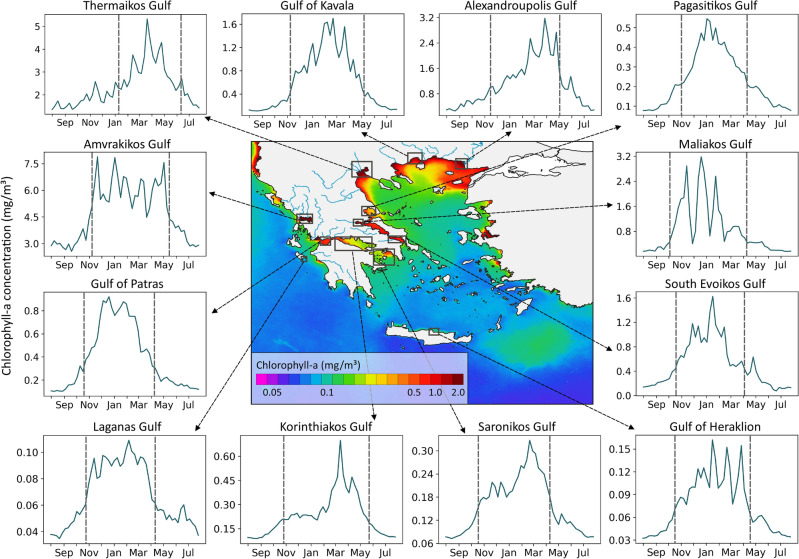
Climatologies of Chl-a concentration (mg/m^3^) time series (1997–2020) in coastal regions of the EMS. The vertical grey lines represent the timings of initiation and termination identified by the phenology algorithm. Central map: Climatology of Chl-a concentrations from January to March for the period 1997–2020. (Plots created using QGIS 3.16 software, https://www.qgis.org/en/site/index.html).

**Figure 4:**
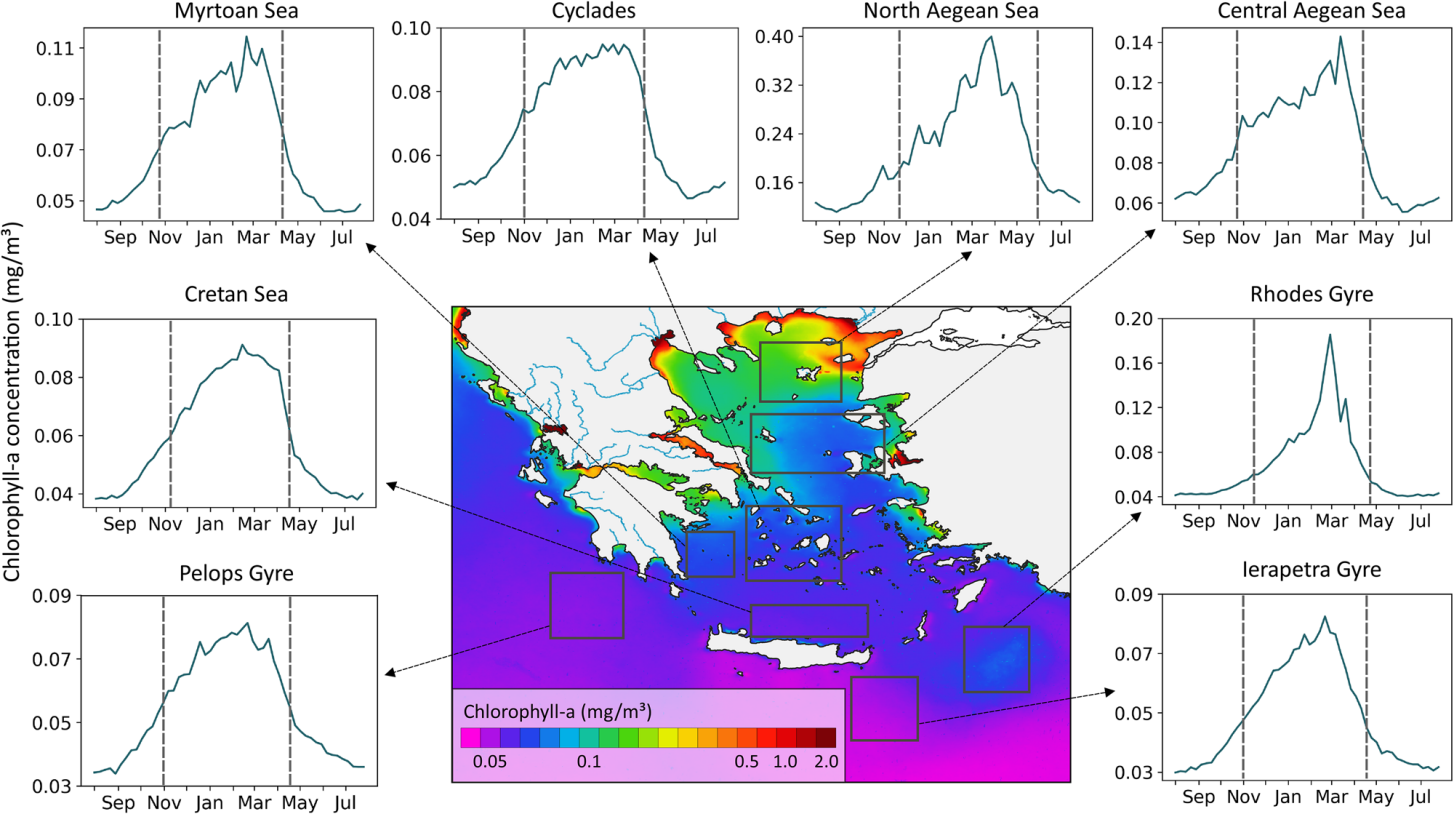
Climatologies of Chl-a concentration (mg/m^3^) in open sea regions in the EMS. Central map: 23-years climatological mean map of the chlorophyll concentration. The vertical grey lines represent the timings of initiation and termination identified by the phenology algorithm. (Plots created using QGIS 3.16 software, https://www.qgis.org/en/site/index.html).

Moreover, since phytoplankton phenology is highly constrained from local prevailing conditions, a further analysis and cross-validation with the available in situ data was conducted in several (i.e., twenty) coastal and open water regions in the EMS. In particular, we highlight and discuss certain key characteristics and phenology indices for selected regions in the Ionian and the Aegean Sea (Figs. 3 and 4). These mean phenology metrics (i.e., initiation, peak, termination, duration, Chl-a mean and maximum values) are summarized in Table 1. Although, the employed in situ Chl-a measurements (Table 2) were sampled at specific time, location and depth, and thus cannot be directly compared to satellite-derived climatologies, there were overall in-line with the derived seasonal patterns, indices and phenology cycles.

### Phenology indices in coastal areas and Gulfs

The coastline of Greece, surrounded by the Aegean, Ionian, and Levantine Seas, receives nutrient-rich waters from small rivers, water outfalls, runoff, and/or various agricultural and industrial activities. This excess nutrient load occasionally leads to large Harmful Algal Blooms (HABs), especially in the Gulfs of Saronikos, Evoikos, Pagasitikos, Amvrakikos, Thermaikos, and the Gulf of Kavala^32^.

Summarizing the results, Chl-a concentration in the selected coastal water bodies ranged between 0.03–7.9 mg/m^3^. The most productive waters (max Chl-a 3.0–7.9 mg/m^3^ and climatology mean 1.0–4.8 mg/m^3^, as shown in Table 1),where prolonged phytoplankton growth periods are reported, occur at the Amvrakikos, Alexandroupolis and Maliakos Gulfs ($\sim$189–203 days). Thermaikos Gulf seems to be the second most productive among the coastal regions, however, it has the shortest growth period duration ($\sim$154 days). The Gulf of Kavala and South Evoikos appear to be mesotrophic (max Chl-a <1.7 mg/m^3^ and climatology mean <1 mg/m^3^). Laganas gulf and Heraklion Bay seem to be the least productive areas (max Chl-a <0.16 mg/m^3^ and climatology mean <0.1 mg/m^3^), under the direct influence of the South Ionian and Cretan Sea oligotrophic open waters respectively Although Chl-a values in the Gulf of Corinth are much lower, this area has the longest phytoplankton growing period.

Eutrophication assessment^33,34^ according to the average Chl-a concentration (MeanChl-a in Table 1) of each coastal area reveals that: (i) Amvrakikos and Thermaikos are characterized as in bad eutrophication status (Chl-a > 2.21 mg/m^3^, (ii) Alexandroupolis, Maliakos Gulfs and Gulf of Kavala in poor status (2.21 > Chl-a > 0.6), (iii) South Evoikos Gulf in moderate status (0.6 > Chl-a > 0.4), (iv) Gulf of Corinth, Patras, Pagasitikos and Saronikos Gulfs in good status (0.4 > Chl-a > 0.1) and (v) Laganas Gulf and Heraklion Gulf in high eutrophication status (0.1 > Chl-a)^35^. A prominent peak suggests that phytoplankton growth is primarily driven by seasonality, whereas the presence of multiple peaks may indicate additional, (potentially random) nutrient inputs occurring during the year, such as domestic or industrial sewage, agricultural runoff, or aquaculture^36^. Even though the climatology time series in the Gulf of Heraklion may exhibit variations that resemble a multi-peak pattern, it’s important to emphasize that these variations are within a significantly narrower range of Chl-a concentrations compared to the gulfs of Maliakos and Amvrakikos. In the cases of Alexandroupolis, the Gulf of Kavala and Thermaikos where multiple peaks occur during the growth perid, the combined effect of river inflows and nutrient rich water masses from the North Aegean Sea is highlighted, where Black Sea Waters (BSW) enter the area through the Dardanelles strait (see details below in the North Aegean section). The impact of BSW fades from east to west along the Thracian coast. The observed delay in the growth period initiation of Thermaikos can be explained by the southerly winds, which initiate to prevail in the region after March, and induce circulation patterns that favour phytoplankton growth, especially in the northern part of the Gulf^37,38^. The inner part of South Evoikos is also another productive area with high anthropogenic pressures and a narrow opening to the Aegean Sea, limiting the water mass exchange with open waters^39^.

In the following subsections, we describe the climatological seasonal cycle of phytoplankton growth and the main phenology metrics for each region. The average Chl-a concentrations during peak season (January to March) are demonstrated in the central map of Fig. 3 to indicate the most productive regions. In addition, to further support the satellite-derived results, we compare our results with in situ Chl-a observations obtained from past scientific cruises as reported in the available literature.

#### Thermaikos Gulf

Thermaikos Gulf is located in the NW Aegean Sea and the inner part hosts the natural harbor of the city of Thessaloniki. The Gulf is divided into three sectors, from Thessaloniki bay towards the inner and then the outer part of the Gulf, representing a north to south gradient of decreasing anthropogenic pressures. The average depth is less than 50 m^40^ and nutrient-rich waters flow in from four rivers, Axios, Aliakmon, Loudias and Gallikos^41^. Maritime traffic, fisheries and heavy industrial operations are only some of the pressures the Gulf receives. Axios river is considered one of the most polluted rivers of Greece affecting the biochemical properties of the Gulf^42,43^.

The initiation of the phytoplankton growth period in the inner Thermaikos Gulf begins between the 9th and 15th of January (Fig. 3 and Table 1). Supporting our analysis, higher in situ Chl-a concentrations were observed based on monitoring surveys within the Gulf (Table 2) in March–May and sometimes in October–November. This second growth period is also depicted by the climatology analysis in Fig. 3. According to the satellite-derived climatology, Chl-a concentration exceeds 5 mg/m^3^ during mid/late March. This is supported by the in situ observations (Table 2), which report that in 1997–2000 Chl-a maxima reached 8.34 mg/m^3^, whereas during the next 15 years Chl-a maxima decreased to 4.52 mg/m^3^ (for Thessaloniki bay, Thermaikos Gulf on average). This decrease in Chl-a levels has been associated with a 32% decrease of Axios river inflows and the removal of $\sim$80% nitrogen from treated urban sewage discharged in Thermaikos^40^. The timing of phytoplankton growth termination occurs in the middle of June (i.e., 11–17th June), resulting in a slightly shorter duration (i.e., $\sim$150 days or 5 months) compared to the rest of the coastal regions under study.

This region appears to have one of the most productive coastal zones, particularly in the inner region and west coast, which are influenced by four river deltas, as well as the Pinios river delta in the south. The same pattern appears for the Chl-a climatology (Fig. 4, central map). Our findings confirm previous studies, which consider the inner Gulf as more eutrophic, whereas trophic conditions improve in the outer Gulf due to fewer pressures, but also due to water masses exchange with the Aegean Sea^44^. Riverine waters are known to move along the west coast of Thermaikos to the south, whereas Aegean water masses enter the Gulf along the east coast from deeper layers in a cyclonic rotation towards the bay of Thessaloniki^45^. Higher productivity and eutrophication frequently leads to large phytoplankton blooms and HABs in almost every season^46–48^. The main HABs species in Thermaikos is the toxic dinoflagellate *Dinophysis acuminata*, which can expand in different parts of the Gulf, but it proliferates mainly in Thessaloniki bay and the deltaic area^44,49^.

**Table 1:** Satellite-derived phytoplankton phenology indices in 20 different regions of the EMS based on 23 years (1997–2020). The max and mean Chl-a concentrations are calculated from the climatology time series.

Site	Initiation	Peak	Termination	Duration(days) (days)	MeanChl-a (mg/m^3^)	MaxChl-a (mg/m^3^)
Coastal						
Amvrakikos Gulf	07–13 Nov	02–08 Jan	14–20 May	189	4.744	7.835
Thermaikos Gulf	09–15 Jan	19–25 Mar	11–17 Jun	154	2.373	5.332
Alexandroupolis Gulf	14–20 Nov	26 Mar–01 Apr	04–10 Jun	203	1.117	3.173
Maliakos Gulf	03–09 Oct	19–25 Dec	09–15 Apr	189	1.117	3.181
Gulf of Kavala	07–13 Nov	20–26 Feb	07–13 May	182	0.614	1.701
South Evoikos Gulf	17–23 Oct	16–22 Jan	02–08 Apr	196	0.516	1.625
Gulf of Patras	17–23 Oct	19–25 Dec	09–15 Apr	175	0.394	0.921
Pagasitikos Gulf	31 Oct–06 Nov	02–08 Jan	09–15 Apr	161	0.234	0.546
Gulf of Corinth	24–30 Oct	12–18 Mar	21–27 May	210	0.230	0.699
Saronikos Gulf	17–23 Oct	20–26 Feb	09–15 Apr	175	0.159	0.327
Laganas Gulf	24–30 Oct	06–12 Feb	09–15 Apr	168	0.089	0.109
Gulf of Heraklion	17–23 Oct	16–22 Jan	16–22 Apr	182	0.075	0.162
Open Sea						
North Aegean Sea	21–27 Nov	26 Mar–01 April	28 May–03 Jun	189	0.212	0.399
Central Aegean Sea	24–30 Oct	12–18 Mar	09–15 Apr	168	0.088	0.143
Myrtoan Sea	24–30 Oct	20–26 Feb	09–15 Apr	168	0.071	0.114
Rhodes Gyre	14–20 Nov	27 Feb–04 Mar	23–29 Apr	161	0.067	0.185
Cyclades	31 Oct–06 Nov	27 Feb–04 Mar	09–15 Apr	161	0.069	0.095
Cretan Sea	07–13 Nov	13–19 Feb	16–22 Apr	161	0.060	0.091
Pelops Gyre	31 Oct–06 Nov	20–26 Feb	16–22 Apr	161	0.055	0.081
Ierapetra Gyre	31 Oct–06 Nov	20–26 Feb	16–22 Apr	161	0.049	0.082

#### Amvrakikos Gulf

The Amvrakikos Gulf, located on the NW coast of Greece is one of the largest semi-enclosed embayments in Greece. It is one of the most important lagoonal complexes, protected by the convention of Ramsar. Water renewal, a vital process for such areas, is carried out via a narrow channel that creates a connection with the Ionian Sea. Amvrakikos Gulf receives discharges from the Arachthos and Louros rivers, which are rich in phosphates. Other sources of nutrient pollution include agriculture, fish farming, and urban sewage^50,51^.

The winter phytoplankton growth period starts between 7–13th November and ends between 14–20th May (Fig. 3 and Table 1). Several peaks are observed in the Chl-a climatology graph, highlighting the complexity of phytoplankton seasonality, while the highest Chl-a value occurs between the 2–8th of January. This multi-peak pattern is evident in the climatological seasonal cycle of Maliakos Gulf (Fig. 3), and is further documented by in situ Chl-a values. Amvrakikos and Maliakos Gulfs are the only two of the studied coastal regions with very limited water masses exchange with the open sea and shallow depths, causing a quite perturbed coastal environment. Therefore, not only river inflows may determine phytoplankton growth in such environments, but also urban and industrial sewage, agriculture runoffs and aquaculture, as documented by Varkitzi et al.^36^. Maximum Chl-a concentration is almost 8 mg/m^3^ in January in Amvrakikos, reaching the highest levels among the studied coastal regions. Higher in situ Chl-a levels have been also observed in March–April, reaching 4.3 mg/m^3^ (Table 2). Amvrakikos is among the EMS coastal areas with frequent HAB events. Water discolourations are often reported due to dinoflagellate HABs mostly, which can last for even three weeks^48,52^.

#### Gulf of Patras

The Gulf of Patras is a semi-enclosed, shallow marine embayment on the NW coast of Peloponnese. The Gulf is the link between Gulf of Corinth and the Ionian Sea while two main rivers (Acheloos and Evinos) discharge into the area. The pollution load transferred by the rivers to the Gulf seems to differ from its adjacent pelagic environment with relatively higher mean Chl-a values (Fig. 3, central map). These regions appear to show relative nutrient enrichment for the development of phytoplankton which can be attributed to anthropogenic pollution, such as urban effluents discharged from the city of Patras, as well as river runoff and bottom enrichment^53^.

In the Gulf of Patras, phytoplankton growth is estimated to initiate in the period 17-23rd October, lasting until early/mid-April. Chl-a maximum concentration (0.92 mg/m^3^) occurs from 19–25th December, earlier compared to regions that presented similar seasonal cycle duration ($\sim$175 days). In situ Chl-a concentrations in 2004–2005 (Table 2) ranged from 0.41–0.54 mg/m^3^ in March (mixing period), and well below 0.16–0.24 mg/m^3^ during the period of thermal stratification (September). During 2012–2015 Chl-a concentrations showed highest chlorophyll values in March 2014 (0.16–0.31 mg/m^3^) and lower in September 2014 (0.15–0.22 mg/m^3^), confirming the previous surveys (Table 2)^54^. The aforementioned Chl-variations are similar to the trends described in the climatology graph in the present study (Fig. 3).

#### Gulf of Corinth

Gulf of Corinth is a deep, semi-enclosed basin, characterized as one of the 26 Important Marine Mammal Areas for the Mediterranean Sea^55^.The Gulf is connected to the Ionian Sea with the Rion-Antirion strait and with the Aegean Sea via the narrow Corinth Canal. The morphology of the northern margin of the Gulf consists of a wide shelf in Antikyra Bay, which passes through a steeping slope and ends to a deep basin floor at 900 m water depth. It is noteworthy, that very few scientific data for Gulf of Corinth are available. The study region is affected by agricultural activities in the coastal zone and pollutants from domestic and industrial effluents, such as aluminum processing plants^56^. On the northern coast, 17 fish farms are located producing mostly sea bass (*Dicentrarchus labrax*), and gilthead seabream (*Sparus aurata*)^57^.

The timing of initiation is usually around 24–30th of October and peak chlorophyll concentration ($\sim$0.7 mg/m^3^) is reached in mid-March (i.e., 12–18th), later compared to the two neighbouring gulfs, Gulf of Patras and Saronikos (Fig. 3 and Table 1). The phenology atlas reveals an important variation of the initiation timings in the Gulf. Only in the coastal zone, the growing period starts in October while in the center of the Gulf it is delayed approximately by two months (i.e., late December to early January (Fig. 2a). During monitor surveys, in situ Chl-a were observed in mid-March (spring) (0.53 mg/m^3^) while in September they decreased by about half (0.15 mg/m^3^). During the years 2004–2005, Chl-a peak was recorded in May (0.21 mg/m^3^) while the lowest value in December (0.09 mg/m^3^)^58^. In 2013–2015 Chl-a concentrations showed high Chl-a concentrations were also recorded in late Spring (March–April) at the coastal areas of the northern part, such as Itea Bay (0.75 mg/m^3^), Antikyra Bay (0.68 mg/m^3^), and southern part (0.504 mg/m^3^)^54^. The increased Chl-a values are related to the relatively increased concentrations of nutrients (i.e., nitrate concentrations), due to anthropogenic pollution from the rivers and/or enrichment from the seabed^54^. Chl-a distribution in the climatology map (Fig. 4) reveals lower concentrations in the open water part of the Gulf, where the depths are greater. The termination of the growing period seems consistent, generally taking place in the time period between the 21st and 27th of May.

#### Saronikos Gulf

The Saronikos Gulf with an area of 2600 km^2^, is situated in the west-central region of the Aegean Sea and is influenced by its water masses^59^. The region is affected by the Athens metropolitan area and is characterized by intensive navigation, tourism and well-developed commercial, and recreational fisheries^60^. The Elefsis Bay (a shallow semi-enclosed area to the north of the Gulf) is considered as one of the most polluted sub-regions on the coastal zone due to industrial activities (oil refineries, shipyards, chemical plants, metal, cement industries, etc.)^61^. Domestic and industrial effluents are released in Saronikos Gulf, while after 1994 the Psitallia Wastewater Treatment Plant is operating, primarily treating before discharging the sewage in near-bottom layers.

The seasonal cycle in Saronikos Gulf is characterized by low Chl-a, with values ranging between 0.07 and 0.33 mg/m^3^ (Fig. 3 and Table 1. Chl-a increases in mid/late–October (17–23th) and the highest values are observed in the middle of winter (i.e., 20–26th February) (Fig. 3 and Table 1). The northern part of the Gulf, close to Elefsis Bay, tends to enter the growth period one week earlier (i.e., 10–16th October) compared to the outer Saronikos Gulf (Fig. 2a). Timings of termination follow a north–south gradient, with Chl-a decreasing later in the northern part and earlier in the south (Fig. 2). In Saronikos Gulf the biomass and abundance of phytoplankton are characterized by elevated seasonal variability. Seasonal patterns are linked to variations in hydrological features^62^ and changes in nutrient concentrations:^27^peaks in biomass occur in spring (March–April) after the replenishment of surface waters with nutrients owing to the typical winter water-column mixing. After spring, the overall phytoplankton biomass decreases while vertical distributions of Chl-a change drastically, displaying deeper maxima in the water column. The environmental quality of Saronikos Gulf is investigated since 1987, providing continuous information about its ecological status. For instance, in Psittalia island, in situ Chl-a ranged from 0.19 mg/m^3^ to 0.67 mg/m^3^ for the period 1998–2015. Regarding the inner Saronikos Gulf, measured Chl-a values ranged from 0.06 mg/m^3^ (July) to 1.09 mg/m^3^ (end March), as well as a second maximum was recorded at the end of November (0.45 mg/m^3^)^26,27,44^.

#### Gulf of Heraklion

The Gulf of Heraklion is on the coastal front of Heraklion city, the administrative capital of Crete Island. The Heraklion harbor is situated there and constitutes one of the most important and active ports in the Eastern Mediterranean, characterized by heavy maritime traffic. Chl-a concentration in the Gulf is estimated to range from 0.03 to 0.16 mg/m^3^, substantially lower than the rest of the studied coastal regions (Fig. 3). The seasonal phytoplankton growth seems to begin between 17–23rd October and peaking time is usually around 16–22nd January. Termination generally occurs 16–22nd April, resulting in an average cycle duration $\sim$180 days (Fig. 3 and Table 1).

In situ concentrations of Chl-a (Table 2) range at low values, but slightly higher compared to the extracted climatology results. Moreover, from bimonthly time series surveys at selected coastal stations in Heraklion Bay similar ranges were obtained: a) 0.05–0.57 mg/m^3^, with peaks again in March–April and lowest values in July (period July 1994–April 1998)^31,63^ , b) 0.03–0.24 mg/m^3^, with peaks in March–April and minima in July, at the POSEIDON HCB station from 2016 to 2021^31^. However, a higher temporal frequency (bi-weekly) sampling conducted in a shallow area (60 m) at the NW of the Gulf of Heraklion from February to May 2005, confirms the satellite-derived phenology assessment of this study, i.e., maximum values recorded in February (0.34 mg/m^3^) while gradually decreasing thereafter until May (0.11 mg/m^3^)^64^. The latter observation pinpoints the need for higher frequency in situ studies to successfully encompass the critical time points of phytoplankton growth.

#### Maliakos Gulf

The Maliakos Gulf is a semi-enclosed and shallow coastal region in Central Greece that receives the discharges of the Spercheios river and is affected by frequent eutrophication and HAB events^36^. Anthropogenic activities that influence the Maliakos coastal zone include agriculture, sewage, fishing and aquaculture^65^.

The phytoplankton growing period begins around 3–9th October and a maximum Chl-a peak of 3.2 mg/m^3^ is observed from 19th–25th December. The duration of the whole growth period is $\sim$189 days, and terminates during 9–15th April (Fig. 3 and Table 1). Based on in situ data collected between 2014 and 2015, Markogianni et al.^66^ reported that during the wet season (November to May), Chl-a values ranged from 0.15–2.17 mg/m^3^ and presented two peaks in March and November. During the dry season (June to October), Chl-a concentrations ranged from 0.19–2.81 mg/m^3^, and reached a peak of 4.98 mg/m^3^ in September near the surface and close to the estuary. This multi-peak pattern is supported by the climatological seasonal cycle of Maliakos Gulf (Fig. 3). This feature highlights the importance of nutrient inputs not only from the river outflow, but also from domestic/industrial sewage through a spillway/anti-flood canal, streams and other non-point sources dispersed along the coastline of Maliakos Gulf^36^.

Chl-a concentrations and phytoplankton abundances can reach high levels throughout Maliakos Gulf. High nutrient inputs from Spercheios river are quickly dispersed in the Gulf due to the fast mixing and homogenization of water masses^36^. Higher Chl-a concentrations and phytoplankton abundance occur mainly near the estuary, and these seem to decrease towards the open waters. The same authors report frequent HABs (*Pseudo-nitzschia* diatom blooms are most often) in the whole Gulf, mainly associated with lower salinity—and indicative of the strong influence of river inflows. The eutrophication assessment in Maliakos Gulf shows an overall mesotrophic status and moderate ecological quality with some improvement away from the estuary^36^.

#### Pagasitikos Gulf

The Gulf of Pagasitikos is a relatively shallow coastal area on the west coast of the Aegean Sea. It is influenced by agricultural run-off, sewage treatment effluents from Volos city, streams and industrial activity^67^. The Gulf is connected with the Aegean Sea and north Evoikos through the narrow Trikeri channel. Since the early 1980s, a few episodes of mass gelatinous mucilage deposition in the north half of the gulf have been seen, presumably due to anthropogenic eutrophication^68,69^.

The phenology of phytoplankton indicates a growth period which initiates between 31 October–6 November, while maximum Chl-a values of 0.55 mg/m^3^ are reached between 2-8th January. The phytoplankton growth terminates on 9-15th April with a total duration of $\sim$161 days (Fig. 3 and Table 1). A previous study at Pagasitikos Gulf, based on SeaWiFS ocean colour observations^70^ showed that the highest Chl-a values were 0.53 mg/m^3^, supporting our analysis. In situ data collected through field surveys indicated that maximum Chl-a values occur in January and April ($\sim$0.5 mg/m^3^) and reach a minimum in September^67,69^. The same authors report that in situ surface Chl-a can reach higher levels during the same period (i.e., 2.26–3.5 mg/m^3^ in January–April and 0.83 mg/m^3^ in September). A signal of higher Chl-a levels on the western coast was also observed in our study (Fig. 4, central map), which can be associated with the combination of stream discharge, sewage diffusion^71^, and vertical mixing due to the known cyclonic circulation there^72^.

#### Alexandroupolis Gulf

Alexandroupolis Gulf is situated in the inner part of the Samothraki continental shelf in the NE Aegean Sea. Evros river, the most important river discharging into the North Aegean Sea in terms of freshwater supply, flows into the eastern part of the Gulf through a delta, which is protected by the Ramsar and Bern Conventions. The water circulation in the broader region is mainly influenced by the Samothraki Anticyclone, which is associated with BSW outflow through the Dardanelles Strait^73^. The initiation of the main phytoplankton growth period occurs between 14–20th of November and terminates in early June (4–10th). The climatology graph reveals several peaks, with the highest Chl-a value (3.17 mg/m^3^) occurring the last week of March (Fig. 3 and Table 1).

Chl-a samples were collected seasonally from various depths in Alexandroupolis Gulf during May 1997, February 1998, June 1998 and September 1998. Chl-a ranged from 0.05 in September (late summer–autumn) to 0.39 mg/m^3^ during February (late winter–spring). During 2013–2018 in Alexandroupolis Gulf Chl-a ranged from 0.12 mg/m^3^ (May-late spring) to 4.05 mg/m^3^ during March (spring) (Table 2). In addition to the main peak, a secondary reduced peak of 1.56 mg/m^3^ occurs during late autumn–winter (November) that is evident in the climatological seasonal cycle (Fig. 3).

#### Gulf of Kavala

The Kavala Gulf is a semi-enclosed embayment that is connected with the N. Aegean Sea through the Thassos Channel and Thassos Plateau. The Gulf is affected by eutrophication due to harbor and industrial activities, sewage treatment operations, Nestos river discharges^32^, as well as Black Sea waters^74^.

Our results show that phytoplankton growth initiates between 7–13th November, and a maximum Chl-a concentration of 1.7 mg/m^3^ is observed in late February (20–26th). The total duration of the growing period is $\sim$182 days (Fig. 3 and Table 1). In situ Chl-a (Table 2), was found to peak at 1.42mg/m^3^, which is consistent with the climatology time series presented in Fig. 3. In earlier studies, average Chl-a levels were reported at 0.14–0.47 mg/m^3^^75,76^. Although large diatoms have been found to dominate the phytoplankton communities^48,76^, blooms and water discolouration events are quite frequent, mainly due to the dinoflagellate *Noctiluca scintillans*.

#### Laganas Gulf

The Laganas Gulf located on the southern shore of Zakynthos Island in the Ionian Sea serves as a migration destination for loggerhead sea turtles (*Caretta caretta*) to lay their eggs. Since 1999, Laganas gulf has been protected by the National Marine Park of Zakynthos, one of the two established Marine Protected Areas in Greece.

Phytoplankton growth initiates from 24–30th October and terminates during 9–15th April with an average duration of $\sim$168 days. The highest value of Chl-a (0.11 mg/m^3^) is observed in early/mid-February (6–12th) (Fig. 3 and Table 1). In situ measurements (Table 2) revealed that at the western coasts of Zakynthos, maximum Chl-a concentrations ranged between 0.09 and 0.26 mg/m^3^ in March 2014, indicating an overall good to high ecological status^54^.

#### South Evoikos Gulf

The southern part of Evoikos Gulf is a semi-enclosed basin with depth < 120 m. A very narrow passage connects North Evoikos with the South and controls the water mass exchanges.

Timing of initiation doesn’t present any significant variations throughout the gulf and was detected between 17th and 23rd of October. The highest Chl-a concentration (1.6 mg/m^3^) is usually observed from the 16th to the 22nd of January. The growth period ends in early/mid-April, resulting in a total phytoplankton growing period of 196 days (Fig. 3 and Table 1). Climatology time series analysis reveals an overall Chl-a mean of 0.52 mg/m^3^ and a seasonal pattern with higher concentrations during spring period and lower in autumn^75,76^.

### Phenology indices in Open Seas

We investigated phytoplankton dynamics in the oligotrophic EMS (Fig. 4) which is generally characterized by a “non-blooming” regime with low Chl-a variations throughout the year^77,78^. Chl-a levels in the EMS demonstrate a moderate increase during winter-early spring, and low Chl-a levels throughout the rest of the year, similar to subtropical waters^79,80^.

Regarding open water regions, the N Aegean Sea is the most productive; with the longest growth period, highest maximal surface Chl-a values and climatology mean (Table 1). The central Aegean comes as the second most productive region, with means for the growth period and the climatology higher than the rest. The Cyclades plateau follows with the same growth period duration to the Rhodes gyre, despite the lower max Chl-a values and climatology mean (Fig. 4 and Table 1). As we are moving further to the south, the Cretan Sea presents a slightly more oligotrophic character than the Cyclades plateau (climatology mean < 0.07 mg/m^3^). Finally, the anti-cyclonic Ierapetra gyre in the Levantine Sea and the Pelops gyre in the Ionian Sea present the same phenology indices, being among the most oligotrophic regions in the region (climatology mean < 0.06 mg/m^3^, as shown in Table 1, Fig. 4). This gradient of increasing oligotrophy on a north to south axis in the Aegean Sea and then to the Levantine and Ionian Seas is a well-documented regime in the literature^30,81^.

To further support our satellite-derived results, we compare them with available in situ Chl-a observations. In particular, the N Aegean Sea is a well-studied region, with a lot of relevant literature dedicated to it. Indeed, when comparing the phenology indices with in situ Chl-a values from several published studies, there is a good agreement between them. For open water regions in the current study that have limited published research, we utilized previously published Chl-a data to compare with our phenology results. Consequently, the overall patterns of phytoplankton phenology examined in the present study are well in accordance with our previous knowledge on these regions.

The following subsections describe in detail the main phenology indices for each sub-region presented in Fig. 4. The annual mean Chl-a concentrations are also shown in the corresponding map, indicating the more oligotrophic character of the open sea compared to more productive coastal waters. However, few regions in the EMS are identified as “intermittently blooming” areas, namely the frontal area in NE Aegean Sea and the Rhodes cyclonic Gyre, which demonstrate oligotrophic conditions with intervals of high Chl-a concentrations as a function of strong physical forcing.

#### North Aegean Sea

The N. Aegean Sea is one of the most productive and important fishing grounds in the EMS, with complex bathymetry, extended shallow continental shelf which alternates with deep basins, and freshwater inputs from the major rivers in the surrounding area^43,82–84^. N Aegean is also adjacent to the Dardanelles Straits through which it receives the colder and fresher Black Sea Water (BSW), connecting the EMS with the Black Sea^73,85^. The circulation of BSW in the wider area is regulated by the temporal variability of BSW flux and the dominant cyclonic circulation of the Aegean Sea^86^ as well as the permanent Samothraki anticyclone that flows around the Island of Samothraki and entraps the inflowing BSW, thus increasing their residence time in N Aegean^87^. The nutrient rich and less saline (less dense) alters BSW flows into NE Aegean Sea and forms a distinct surface layer of 0–20 m or 50 m thickness, under which the warmer and saltier (denser) water of Levantine origin is detected, forming a dynamic thermohaline front^73,85^. BSW affects directly the NE Aegean, but it can disperse in a wider area of the N Aegean following known hydrological patterns^88^. The inflowing BSW carries high loads of particulate and dissolved organic matter (POM, DOM) and less inorganic nutrients^82,89–91^ which favour autotrophic/heterotrophic biomass and primary/secondary production^30,81,83,92,93^.

Phytoplankton growth period initiates in late November (i.e., 21–27th) and terminates in late May–early June, with a total duration of $\sim$189 days (Fig. 4 and Table 1). This suggests that the blooming period in the North Aegean Sea shows the longest duration among the studied open waters. Maximum Chl-a values of 0.4 mg/m^3^ are detected from 26th March to 1st April, and the highest climatology Chl-a average of 0.21 mg/m^3^ for the whole study period, compared to all other open water regions (Table 1). Previous in situ surveys in N Aegean confirm that Chl-a peaks within the surface layer (0–20m) during March–April^30,81,94,95^. The same studies report similar high in situ Chl-a levels (ranging at 0.34–0.98 mg/m^3^) with our results during March–April.

#### Central Aegean Sea

The Central Aegean is known to be a transient area between the North Aegean, which is directly affected by the productive BSW, and the South Aegean (Cyclades plateau and Cretan Sea), which is influenced more by the oligotrophic Levantine waters. The phytoplankton growth period at the Central Aegean Sea commences slightly earlier (i.e., in late October) and terminates in early/mid-April. However, the initiation and termination in Central Aegean precede by almost a month those in N Aegean. Moreover, the total duration is 168 days and therefore, the growth period in Central Aegean is two weeks shorter than in N Aegean (Fig. 4 and Table 1). The highest Chl-a values of 0.143 mg/m^3^ are observed during 12–18th March, whereas the growth period mean is 0.112 mg/m^3^ and the climatology mean is 0.088 mg/m^3^ (Table 1).

The phenology metrics are comparable to those obtained from on-site measurements, however, there are limited studies on phytoplankton in this region of the Aegean Sea^30,81,96^. Therefore, we analyzed in situ samples that have been collected in past cruises (Table 2).In situ Chl-a ranges (Table 2) from 0.02 up to 0.31 mg/m^3^ within the surface layer (0–20 m), with Chl-a peaking during February–March (mean Chl-a 0.163 mg/m^3^ for this period). A quite similar in situ Chl-a mean is reported also from the deeper layer 0–50 m (0.16 mg/m^3^ during February–March). First optical depth during these months, as estimated from satellite-derived observations, is approximately 24–25 m on average. High spring Chl-a levels are known to exist in deeper layers of the water column as a result of deep winter mixing in the area^30,81^. By further assessing the mean phenology indices, the low levels of annual Chl-a mean (< 0.1 mg/m^3^) in Central Aegean are confirmed (Table 1), indicating the oligotrophic character of the considered region.

#### Myrtoan Sea

The Myrtoan Sea plays an important role in the water circulation and exchange^97^ and is characterized by the permanent or recurrent Myrtoan cyclone. It is a relatively shallower region reaching 1000 m at the deepest spots^88^.

The phenology algorithm indicates that Chl-a values begin to increase during the last week of October and peak (0.11 mg/m^3^) in late February. The growing period terminates in early/mid April resulting a total duration of $\sim$168 days (Fig. 4 and Table 1). According to in situ measurements in September 2020, Chl-a ranged from 0.01 mg/m^3^ at the surface layer to 0.19 mg/m^3^ at 50m depth, which is close to our results(Table 2). The first optical depth as estimated from satellite observations was $\sim$40 m during this period. Dimiza et al.^98^ also reported similar Chl-a values during late February 2008 (0.195 mg/m^3^), indicating the presence of the phytoplankton bloom(Table 1).

#### Rhodes Gyre in the Aegean Sea

The Rhodes Gyre is a permanent cyclonic gyre in the general circulation of the Levantine Basin in the EMS.^99,100^. Despite being situated in the Mediterranean’s most oligotrophic basin, it has relatively high primary productivity, linked to the formation of Levantine Intermediate Water^101^, affecting the distribution of nutrients, the biological activity and the mesozooplankton communities^102^. The phenology of phytoplankton observed at this region is characterized by a growing period occurring between November (14–20th) and April (23–29th). The total blooming period lasts $\sim$161 days and the maximum Chl-a concentration values of 0.185 mg/m^3^ are recorded in late February-early March (Fig. 4 and Table 1).

In situ measurements obtained from scientific cruises^103,104^ revealed that Chl-a concentrations in Rhodes gyre show a seasonal pattern with maximum values during late winter-early spring (0.12–0.47 mg/m^3^) and minimum during late summer-early autumn (0.05–0.16 mg/m^3^), which is consistent with our results. A recent study by D’Ortenzio et al.^99^, based on Argo-floats, in situ and satellite data, indicated a similar phytoplankton growing period initiating during late November and ending in late April. In early March, a biomass peak was observed (Chl-a $\sim$0.6 mg/m^3^) as indicated by in situ HPLC data, confirming that the phytoplankton increase is affected by mixed layer depth and nutrients dynamics related to winter mixing. Past satellite-based studies by Pedrosa-Pamies et al.^105^ and models simulations implemented by Napolitano et al.^106^ also confirmed that phytoplankton bloom occurred in March (with Chl-a values 0.4 mg/m^3^), while the lowest Chl-a values were recorded during summer (Chl-a 0.07 mg/m^3^). Due to nitrates consumption, phytoplankton exhibits a weaker bloom during mid-December to mid-January^106^. A weaker peak in January is also demonstrated in climatologies Fig. 4. It is evident that in Rhodes gyre region, strong over-turning episodes during winter and atmospheric conditions occasionally result in nutrients pumping into the euphotic zone, leading to unusually high increase of biomass (Chl-a values > 1 mg/m^3^) and primary production rates^106^.

#### Cyclades plateau in the Aegean Sea

The Cyclades archipelago forms a shallow plateau (less than 340 m depth) that separates the Cretan Sea (S Aegean) from the North Aegean; two basins with considerably different hydrographic characteristics shaped by the influence of oligotrophic Levantine Sea waters and more productive BSW, respectively^107^. Winter convective mixing in the broader area of Cyclades and the S Aegean are known to drive the water column overturn^82^, causing nutrient enrichment of the euphotic zone and consequently higher phytoplankton biomass production^30,81,96^. According to these hydrographic features, the Cyclades plateau is a generally oligotrophic region.

Phytoplankton growth is estimated to initiate during 31st October to 6th November and terminate during 9th to 15th April. The duration lasts $\sim$161 days, which is the Cretan Sea and the Rhodes, Ierapetra and Pelops gyres. The highest Chl-a values (0.095 mg/m^3^) are observed during 27 February–5 March. Lower Chl-a levels are found on average for the phytoplankton growth period (0.087 mg/m^3^) and overall during the whole seasonal cycle (0.069 mg/m^3^) (Fig. 4 and Table 1).

Due to the scarcity of previous studies in the Cyclades plateau^30,96^, the comparison of phenology results with in situ Chl-a variations is quite limited. Therefore, we processed sparse in situ Chl-a data from previous campaigns from the region (Table 2), which confirm the climatology seasonal pattern (with higher values in late winter-early spring and lower in summer), revealing a Chl-a range from 0.04 up to occasionally 0.45 mg/m^3^ on an annual basis. In accordance with our results, the highest in situ Chl-a values were recorded in the surface layer (0–20 m) during February–March, presenting mean values of 0.23 mg/m^3^. High in situ Chl-a values are also reported from deeper layers during February–March (0.23 mg/m^3^ at 0–50 m and 0.22 mg/m^3^ at 0–200 m), which is a known pattern generated by the deep winter mixing of the water column^30,81^, as for the case of Central Aegean mentioned previously. A stronger than ordinary convection event in Cyclades and the broader South Aegean happened in winter 2008, which homogenized the water column profoundly^108^. Consequently, an unprecedented spring bloom was documented for the first time in the Cyclades plateau during the end of March-beginning of April, with Chl-a maxima of 0.52 mg/m^3^ at 100 m and 0.37 mg/m^3^ at 150 m^30^.

#### Cretan Sea in the South Aegean Sea

The Cretan Sea covers the southern part of the Aegean Sea and links the Levantine Basin to the Ionian Sea. The hydrological structure in the Cretan Sea is dominated by multiple scale circulation patterns and intense mesoscale activity. The system is largely driven by the regional hydrodynamics and the occurrence of deep mixing events^109^. The water circulation is dominated by a succession of anticyclonic (west) and cyclonic (east) eddies forming a west–east dipole^110,111^.

According to the phenology metrics produced in this study, phytoplankton growth period begins between 7–13th November (Fig. 4 and Table 1). Maximum values of Chl-a and primary production occur in late winter-early spring associated with favourable hydrological conditions and increased diatom abundance, while minimum concentrations occur in late summer^63^. The maximum Chl-a value according to the climatology time series is recorded in mid-February, reaching 0.09 mg/m^3^. Gotsis-Skretas et al.^112^ analyzed the vertical distribution of Chl-a with data collected during four seasonal cruises undertaken from March 1994 to January 1995 and reported the winter and spring as intense mixing periods that result in enhanced phytoplankton growth; a temporal trend that is also captured by the present analysis. Similar observations with higher temporal resolution were obtained during a bimonthly monitoring along an offshore gradient between July 1994 and October 1995 (Table 2), yielding maximum Chl-a values in March–April (0.21–0.39 mg/m^3^) and minimal in July (0.04–0.05 mg/m^3^)^63^. At interannual time scales, the temporal dynamics of phytoplankton growth resemble more those revealed by the phenology analysis in this study, with maximum values recorded earlier, between January and March (0.18–0.24 mg/m^3^) and lowest values in June–July (0.033 mg/m^3^). The latter data are incorporated into a compilation of data over 34 years (1987–2020) of phytoplankton studies in the Aegean Sea^31^, which reveal two peaks, in January and March (0.28 and 0.30 mg/m^3^, respectively) slightly deviating from the unimodal phenology pattern estimated in this study.

#### Pelops Gyre in Ionian Sea

The Ionian Sea connects the EMS and the Adriatic Sea. In the south Ionian Sea, the Pelops gyre subsists as an intense anticyclone with variations in shape and dimensions^111,113^. Its interannual variability is strongly affected by the Atlantic Ionian Stream (AIS)^114^.

The climatology time series reveal that the lowest Chl-a values are observed during summer. The phytoplankton growing period initiates during late October early November (31st October–6th April), and it ends in mid/late April (16–22 April) (Fig. 4 and Table 1). This is confirmed by the analysis of Lavigne et al.^115^ who investigated the inter-annual variability of phytoplankton phenology from 1998 to 2012 in the Ionian basin. Within the Pelops gyre, Chl-a concentration peaks (0.08 mg/m^3^) between 20–26th February, whereas the growth period mean is 0.07 mg/m^3^ and the climatology mean is 0.055 mg/m^3^. Moreover, Pelops’ Chl-a levels during the peak period are ranked among the lowest. This is in accordance with previous knowledge upon which the Ionian presents lower Chl-a and particulate organic matter concentrations compared to the Aegean Sea during March–April^30,96^.

After processing of in situ Chl-a data (Table 2), Chl-a concentrations ranged from 0.016 to 0.169 mg/m^3^ in the surface layer (0–20 m), which are relatively close to our results. Indeed, the maximal in situ values were also found during February–March, with 0.04 mg/m^3^ at the surface layer (0–20 m) and increasing Chl-a values in deeper layers (0.055 mg/m^3^ at 0–50 m and 0.06 mg/m^3^ at 0–200 m depth). This vertical pattern constitutes the formation of the Deep Chlorophyll Maxima (DCM), which is a permanent characteristic of the Ionian Sea^78^. The Ionian, together with the Levantine Sea, occupy the most oligotrophic and transparent waters in the Mediterranean. Salgado-Hernanz et al.^18^ support that in the Ionian Sea only a very low increase of Chl-a concentration occurs in winter ($\sim$0.1 mg/m^3^), which results in essentially constant Chl-a throughout the year^79^. Varkitzi et al.^30^ reported a clear DCM at 75m in the Ionian, together with very low levels of Chl-a and phytoplankton productivity.

#### Ierapetra Gyre in Levantine Sea

In the southeast region of Crete the wind-induced Ierapetra anticyclonic gyre^116^ presents a very strong signature in sea surface height and sea surface temperature^88,117^. The intense anticyclone presents seasonal as well as inter-annual variability^100^. It has been proven to be related to topographic Rossby waves^118^ and possibly enhanced by Etesian winds^117^.

Based on the climatology analysis, the growth period begins during late October–early November and terminates between the 16th and 22nd of April (Fig. 4 and Table 1). The maximum Chl-a value (0.08 mg/m^3^) is observed during late February (i.e., 20–26th), and compared to Rhodes gyre it is much lower, indicating a less productive region. Ierapetra gyre represents the lowest growth period mean (0.06 mg/m^3^) and climatology mean (0.05 mg/m^3^) among all the other open water regions. Karageorgis et al.^119^ revealed that in early-mid April 2016 Chl-a values at surface were relatively low (0.04 mg/m^3^), while the maximum values were found at 75–100 m layer (0.23 mg/m^3^). Varkitzi et al.^30^ also confirmed that low Chl-a (0.077 mg/m^3^) concentrations were, recorded during cruises in Spring and Summer 2008 close to Ierapetra gyre (Levantine sea).

**Table 2:** Sample collection sites, sampling periods and the relevant projects/cruises of the in situ Chl-a concentration datasets.

Site	Chl-a (mg/m^3^)	Year	Project/Cruise
Alexandroupolis Gulf	0.05–0.39	1997–1998	North Aegean–INTERREG project
	0.17–4.05	2013–2018	WFD monitoring
Amvrakikos Gulf	<4.3	2012-ongoing	WFD monitoring
Central Aegean	0.02–0.31	1988-1989	LIA 7, 8, 9 and 10 cruises
		1997–1998	MATER
		1997–2000	INTERREG
		2008	SESAME
Cretan Sea	0.033–0.34	2010–2021	POSEIDON E1-M3A
Cyclades Plateau	0.04–0.45	1988	LIA 7 and 8 cruises
		1994	MATER
		2008	SESAME
Gulf of Heraklion	0.05–0.57	1994–1998	CINCS Project
	0.03–0.24	2016–2021	POSEIDON HCB
Gulf of Kavala	<1.42	2012-ongoing	WFD monitoring
Gulf of Patras	0.16–0.54	2000	MED-POL
		2004–2005	Habitat Assessment Baseline survey in the West Patraikos Licence Area
Myrtoan Sea	0.007–0.191	2020	MARRE-Project
			CRELEV cruise
Pelops Gyre	0.16–0.169	1991	091 cruise
		1991	POEM
		1994	PELAGOS
		2008	SESAME
Saronikos Gulf	0.19–1.09	1987-ongoing	MED-POL and Saronikos monitoring
Thermaikos Gulf	0.11–9.01	1992-ongoing	Thermaikos and WFD monitoring
West Coasts of Zakynthos	0.09–0.26	2014	WFD monitoring

## Conclusions

Here, phytoplankton phenology metrics, based on a satellite ocean colour multi-sensor product, were analyzed in order to describe the phytoplankton seasonality in several coastal and open water regions in the EMS. The proposed phenology algorithm has been developed to detect the main phytoplankton growth period, allowing us to record and investigate its growth timing metrics for the entire study area.

Satellite-derived phenological metrics were compared to corresponding indices derived from in situ Chl-a time series in the Saronikos Gulf. This initial validation analysis confirmed the feasibility of measuring phytoplankton phenology from satellite-based ocean colour data at a weekly resolution in the EMS. Following this, climatologies of Chl-a concentration were presented for twenty selected key regions and compared to available (literature or historical samples) in situ Chl-a measurements obtained through from past scientific cruises. Our findings suggest that the current methodological approach detects quite precisely the seasonal variations and phenological metrics of phytoplankton.

We envisage that the phenology atlas presented here will improve the knowledge on ecosystem functioning, spatiotemporal patterns of phytoplankton biomass (food availability) and potential linkages with higher trophic levels, such as filter feeders (e.g., bivalves and sea cucumbers), zooplankton, commercially-important fish, and marine mammals in the EMS. It may also support efforts directed at water quality management, aquaculture activities in coastal areas^120^, and strategies for HABs monitoring and control^121^. Future analysis could focus on linking the trends of phenology metrics with physical forcing factors and/or oceanic warming, to further understand evident alterations in bloom timing. Since the EMS has been considered as a hot spot for climate change, future work may focus on phytoplankton indices’ sensitivity and response to extreme warming/cooling episodes (Marine Heatwaves and Cold Spells)^122,123^. Further research on the interannual variability of ecological indicators, and linkages with fisheries dynamics^10^ will be also essential for the marine trophic interactions investigation and ecosystem management in the EMS.

## Materials and methods

### Satellite remote sensing

The sea surface Chl-a concentration (mg/m^3^) Level-4 multi-sensor (SeaWiFS, MODIS-Aqua, MERIS, and VIIRS sensors) reprocessed product was obtained from the Copernicus Marine Environment Monitoring Service (CMEMS) at 1-day and 1-km resolution. The dataset covers the period from September 1997 to December 2020 for the Mediterranean Sea. The CMEMS chlorophyll product for the Mediterranean Sea is derived through a four-step process. Initially, the MedOC4 Chl algorithm^124^ tailored for Case I waters is applied to the entire L3 field. Subsequently, the AD4 algorithm^125^ for Case II waters is applied similarly. Pixels are then classified into the two water types or identified if they do not align precisely with either. Discrimination between these types relies on a pixel-by-pixel comparison of the satellite spectrum with average water type spectral signatures, utilizing in situ measurements for both Case I and Case II waters. The applied algorithms improve the accuracy of the final results, particularly for low chlorophyll values, which describe the majority of the open water EMS^126^. The in situ dataset, MedBiOp, aids in selecting pure Case II spectra through k-means cluster analysis^127^. Finally, the two images are merged, incorporating a Mahalanobis distance-based weighting approach. This final dataset provides surface water chlorophyll content approximating one-fifth of the euphotic depth. For more detailed information regarding the dataset and the processing procedures applied, refer to the Product User Manual.

Focused on the eastern part of the Mediterranean Sea, the geographic position of the study area was set between 18.5^∘^E to 30.6^∘^E longitude and 32^∘^N to 42^∘^N latitude. Prior of being temporally averaged, the quality of the satellite-derived Chl-a concentrations was statistically examined. To achieve this, we utilized a sliding window of 10 x 10 pixels, enabling us to spatially average the data and create time series for each individual window. Outliers were identified using a threshold of ± 2 times the median of the data. Any Chl-a concentration that exceeded this threshold on a given date was flagged as anomalous. To confirm the validity of these flagged dates, a visual examination was conducted to detect irregularities or anomalous values in the data. During this process, we consulted with the data producers (CMEMS) and identified 65 daily files, comprising approximately 0.77% of the total data, which predominantly contained interpolation errors. As a result, these files were excluded from the analysis, ensuring the robustness and accuracy of the findings.

### In situ Chl-a data

**Figure 5:**
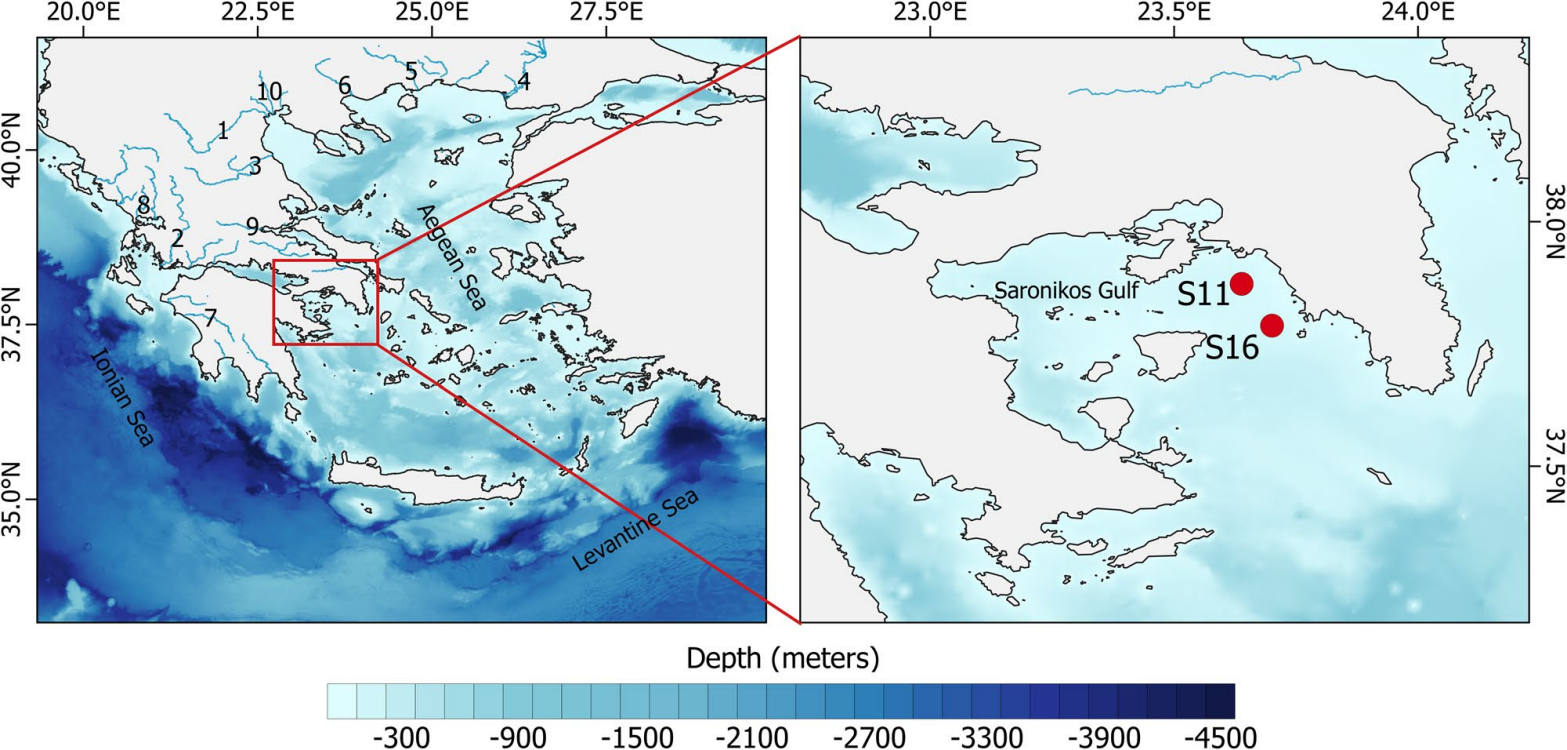
Panel (**a**): Sea-floor elevation of the EMS. Numbers represent the main rivers with 1: Aliakmonas, 2: Acheloos, 3: Pinios, 4: Evros, 5: Nestos, 6: Strimonas, 7: Alfeios, 8: Arachtos, 9: Spercheios, 10: Loudias, Axios, Gallikos (left to right). Panel (**b**): The location of the two sampling stations S11 and S16 (23.64^∘^ E, 37.87^∘^ N and 23.70^∘^ E, 37.79^∘^ N, respectively) in inner Saronikos Gulf. (Plots created using QGIS 3.16 software, https://www.qgis.org/en/site/index.html).

A long-term in situ dataset from the inner Saronikos Gulf was used to evaluate the performance of the phenology algorithm we used. From 1997 to 2017, in situ Chl-a data were obtained in the frame of the program “Monitoring of Saronikos funded by EYDAP SA” and through numerous scientific cruises in two sampling stations; S11 and S16 (Fig. 5). The scientific cruises were conducted every year (in some cases data were collected every month) in order to be as representative as possible for all the seasons, ultimately resulting in 355 samples in total. To assess Chl-a concentrations, seawater samples were collected from discrete depths of the water column (2, 10, 20, 50 and near the bottom) using 10 L Niskin bottles (Rosette sampler adapted to a CTD type instrument SBE-9 or individually on a hydrowire). Sea water was filtered through Whatman GF/F Microfiber filters (1 or 2 liters of sea water, depending on trophic status of each station). The filters were kept deep frozen in dark at − 15 °C and analyzed at the laboratory on a TURNER TD-700 fluorometer according to the method of Holm-Hansen et al.^128^. To further evaluate the satellite-derived results, we used in situ Chl-a observations obtained from past scientific cruises in several coastal and open waters. It is important to acknowledge that these in situ measurements exhibited limitations regarding spatial and temporal coverage, as well as missing or inadequately reported information about sampling locations, depths and time. Consequently, a robust statistical comparison with literature-derived in situ values, integrated with the ocean colour dataset, is not feasible due to the scarcity and lack of necessary details. The corresponding projects and cruises are demonstrated in Table 2.

### Bathymetry

Sea-floor elevation data with a grid resolution of 1/16*1/16 arc minutes have been derived from the EMODnet Bathymetry portal. This portal was initiated by the European Commission as part of developing the European Marine Observation and Data Network (EMODNet).

### Data preprocessing and analysis

The phenology algorithm that was implemented in the current study was based on the cumulative sums of anomalies method, which utilizes a threshold criterion^8,16,22,25,129^. To construct the climatological seasonal cycles, we calculated the weekly means for each 7-day bin over the 23 years spanning the period from 1997 to 2020. This approach was chosen to provide a gap-free annual cycle at every pixel in the EMS. This approach is crucial for accurately calculating the phenology metrics since the cumulative sum of anomalies method requires a complete (i.e. no missing data) Chl-a time series as an input. Even though averaging over 7-days reduces the resolution at which events in the phytoplankton growth period can be estimated, it does not significantly affect the spatial pattern of the estimated phenological metrics^130^. The calendar year was delimited from August to July to ensure that the bloom of interest (i.e., winter-to-spring bloom) is centered in the time series, as shown in Figs. 3 and 4. The threshold criterion was used to estimate the phenological indices of the timing of initiation, peak, termination, and duration of phytoplankton growth, based on the weekly Chl-a climatology time series. In particular, the threshold criterion was defined as the long-term median plus 10% per pixel. Chl-a anomalies were calculated by subtracting the threshold criterion from the climatology, and the cumulative sums were then computed. Lastly, the gradient of the cumulative sums of anomalies was smoothed with a Gaussian filter and employed to identify the desired phenology metrics. In addition to the metrics mentioned earlier, we also calculated the average Chl-a value of the seasonal cycle (i.e., climatology mean or MeanChl-a), which is presented in Table 1.

### Phenology indicators

**Figure 6:**
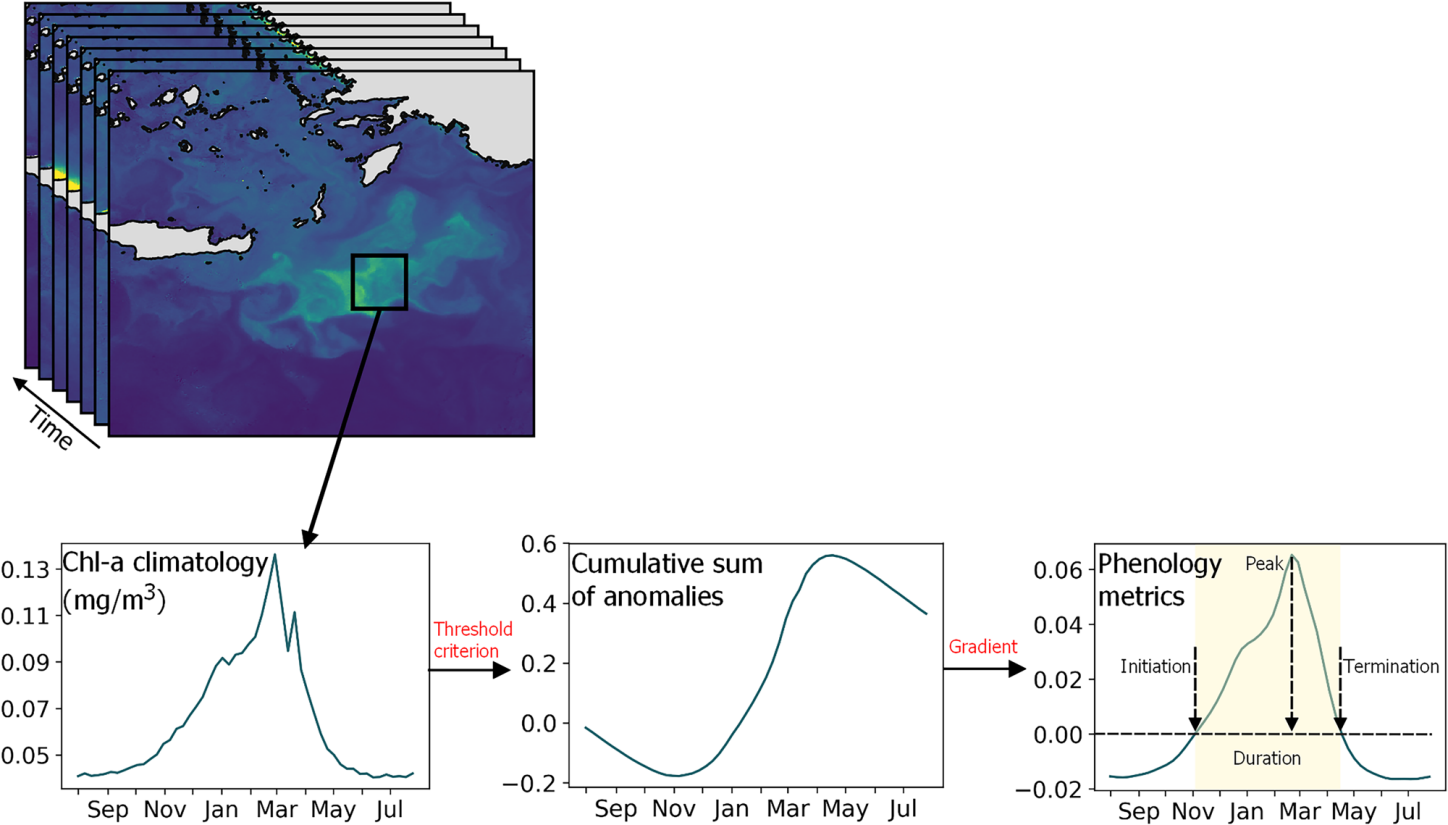
Schematic diagram of the phenology method used to detect the main phytoplankton growth period from Chl-a time series. (Plots created using QGIS 3.16 software, https://www.qgis.org/en/site/index.html).

Different threshold criteria were tested varying from 5% to 20% plus median, before the final choice was made. Choosing a low threshold criterion would detect longer blooming periods and/or multiple secondary blooms. On the contrary, a higher threshold criterion would possibly exceed the higher maximum chlorophyll values within the growing period, resulting in “no-bloom” cases. The choice of threshold is arbitrary, and depends on the type of the analysis (e.g., interannual or seasonal), and several thresholds have been implemented in several regions of the world’s oceans^10,22,25,129,131^. This method is built to detect the characteristics of the main phytoplankton blooming period, without taking into consideration any secondary blooms. The timing of initiation and termination were identified as the times when Chl-a concentration rose above and fell below the threshold criterion (i.e., when the gradient of the time series changed sign). The duration is calculated as the number of 7-day composites between the timing of initiation and termination, while the peak is identified once Chl-a reaches its highest signal. In order to investigate the seasonal cycles of several coastal and open sea regions, we modified the pixel-based approach to accommodate multiple pixels (i.e., slices). This modification allowed us to generate the climatology time series and phenology metrics for each specific area considered. The data averaged in space for deriving these climatology time series and phenology metrics are represented by the black rectangles in Figs. 3 and 4. A schematic representation of the phenology method is shown in Fig. 6.

### Validation of satellite-derived phenology

To validate the outputs of the phenology algorithm with the available in situ datasets, the diffuse attenuation coefficient K_d_(490) was estimated, which represents the light attenuation within the water column. For the K_d_ determination in inner Saronikos Gulf (where the long-term in situ datasets are), we utilized the CMEMS daily L3 K_d_ product with a spatial resolution of 1 km^132–134^. The first optical depth (Z_90_)^135^ in the inner Saronikos Gulf was calculated using the following equation: Z_90_ = 1/K_d_(490). Then, we temporally aligned the daily K_d_ values with daily in situ Chl-a measurements. To obtain Chl-a values at depths from 1 to 75 m with 1 m intervals, we performed a linear interpolation of the in situ Chl-a data. Finally, we estimated Chl-a integrated within the first optical depth using the trapezoidal rule.

For the comparison of phenological indices derived from satellite observations and in situ data, the two datasets were matched in time and space. Using the exact same dates and station locations, the daily Chl-a observations were extracted from the remote sensing data. The weekly (7-day) time series for both datasets were produced and the phenology metrics were calculated as described above (refer to the Phenology Indicators subsection for details).

### Potential biases

While each coastal region may exhibit unique optical characteristics, the good agreement between in situ and satellite-derived phenology metrics serves as a valuable benchmark for establishing the broader utility of satellite-derived Chl-a data for phenology studies. This approach allows us to assess temporal phytoplankton dynamics in regions where in situ sampling is challenging, enhancing the applicability of our findings across various ecosystems.

Outliers can potentially influence the computation of phenology metrics. To mitigate this, we conducted extensive data preprocessing, including outlier detection and removal, as described in the Materials and Methods section. We also recognized the importance of selecting an appropriate threshold, that is aligned with our seasonal analysis and the biological dynamics of the study region.

Additionally, it is well known that satellite-derived ocean colour measurements of Chl-a concentrations, has acknowledged weaknesses (i.e., case II waters, optically complex waters, bottom reflectance in coastal zones), where algorithms over/underestimate the absolute chlorophyll measurements^136,137^. Here we utilized a product that is based on the regionally-tuned Mediterranean algorithm OC4^124^. Regardless of the fact that the MED-OC4 algorithm was employed, it is important to note that it may still exhibit variations in estimating absolute chlorophyll values, particularly in regions situated closer to the coastlines. However, the phenology indices are produced on relative changes and trends of phytoplankton seasonal cycles. Therefore, the phytoplankton growing season is calculated based on variations of Chl-a over time rather than on the absolute Chl-a values. In addition, to verify the phenology outputs, we validated the final results with a long term in situ Chl-a time series.

## Data Availability

The in situ Chl-a dataset used in the present analysis is available on reasonable request. Ocean colour and bathymetry datasets are freely available at https://marine.copernicus.eu/(OCEANCOLOUR_MED_BGC_L4_MY_009_144 and OCEANCOLOUR_MED_BGC_L3_MY_009_143) and https://emodnet.ec.europa.eu/en, respectively.
